# Benefits of Innovative and Fully Water-Compatible Stationary Phases of Thin-Film Microextraction (TFME) Blades

**DOI:** 10.3390/molecules26154413

**Published:** 2021-07-21

**Authors:** Łukasz Sobczak, Dominika Kołodziej, Krzysztof Goryński

**Affiliations:** Bioanalysis Scientific Group, Faculty of Pharmacy, Collegium Medicum in Bydgoszcz, Nicolaus Copernicus University in Toruń, Dr. Antoniego Jurasza 2 Street, 85-089 Bydgoszcz, Poland; lukasz.sobczak@cm.umk.pl (Ł.S.); dominika.kolodziej@cm.umk.pl (D.K.)

**Keywords:** sample preparation, thin-film microextraction, solid phase microextraction, octadecyl, polar end-capped particles, liquid chromatography, TFME, SPME, C18, HPLC-MS/MS

## Abstract

Octadecyl (C_18_) groups are arguably the most popular ligands used for preparation of solid phase microextraction (SPME) devices. However, conventional C_18_-bonded silica particles are not fully compatible with the nearly 100% aqueous composition of typical biological samples (e.g., plasma, saliva, or urine). This study presents the first evaluation of thin-film SPME devices coated with special water-compatible C_18_-bonded particles. Device performance was assessed by extracting a mixture of 30 model compounds that exhibited various chemical structures and properties, such as hydrophobicity. Additionally, nine unique compositions of desorption solvents were tested. Thin-film SPME devices coated with C_18_-bonded silica particles with polar end-capping groups (10 µm) were compared with conventional trimethylsilane end-capped C_18_-bonded silica particles of various sizes (5, 10, and 45 µm) and characteristics. Polar end-capped particles provided the best extraction efficacy and were characterized by the strongest correlations between the efficacy of the extraction process and the hydrophobicity of the analytes. The results suggest that the original features of octadecyl ligands are best preserved in aqueous conditions by polar end-capped particles, unlike with conventional trimethylsilane end-capped particles that are currently used to prepare SPME devices. The benefits associated with this improved type of coating encourage further implementation of microextractraction as greener alternative to the traditional sample preparation methods.

## 1. Introduction

Ever since liquid chromatography moved towards its current leading position among available analytical methods, both sample preparation and chromatographic separation were performed predominantly with octadecylsilane (ODS, C_18_)-bonded particles. Joseph Jack Kirkland, one of the early pioneers in high-performance liquid chromatography (HPLC) stated that “C18-based silanes were readily available at that time and reasonable in cost” [1] when the method was initially developed, and since then, “tradition” paved their way to their present status. Regardless of circumstantial beginnings, C_18_-bonded particles are often superior to any alternative in many applications and are thus likely to remain popular. However, evolution in this field is occurring. Novel supports with improved particle shapes, chemistry, and increased purity; end-capping that mitigates undesired interactions; and incorporating additional functional groups as end-capping agents or as complementary functional ligands are a few of the more recent technological advances in this field of study. Thus, polar end-capped, polar embedded, positively charged surface, or mixed-mode (containing ion-exchanging groups) chemistries are readily available in contemporary octadecylsilane HPLC and UHPLC columns.

For sample preparation, methods such as solid phase extraction (SPE) or solid phase microextraction (SPME) also strongly rely on octadecyl (C_18_) chains as the most popular chemistry that has been successfully used in combination with liquid chromatography. This fact is perhaps best highlighted by the currently available SPME devices that are designed for direct immersion into liquid samples. Such solutions are particularly sought when so-called “green” extraction procedures are desired or during the analysis of thermally unstable and non-volatile compounds that cannot be analysed with gas chromatography. In such cases, C_18_-based coatings are one of only two options that are marketed as SPME devices and are compatible with liquid chromatography; the other option is PDMS/DVB (polydimethylsiloxane/divinylbenzene). SPME is considered a “greener” alternative to concurrent sample preparation techniques. In accordance with the principles of Green Analytical Chemistry, microextraction methods reduce organic solvents consumption, and combine sample collection, extraction and analyte enrichment above required concentration into a single step [2,3,4]. Additionally, the method is characterized by low energy consumption, low laboratory waste production, and device reusability, while still enabling performance comparable or superior to the traditional methods [5]. C_18_-coated SPME tips and fibers are already popular around the world, as indicated by the publications of many research groups within the last 2 years [6,7,8,9]. C_18_ is also one of most popular coating types for the development of new SPME solutions, such as thin-film microextraction (TFME) blades. Although just recently commercialized, the format of TFME had already been shown to be useful in high-throughput analysis of biological fluids due to its physical stability, long-term reusability, and high degree of reproducibility [10,11]. Additionally, TFME is characterized by a greater surface area, which increases the extraction efficacy (yield), and thin layer(s) of the coating that makes attainment of thermodynamic equilibrium of the process easier compared to commercially available SPME tips or fibers. Concurrently, the format of the blades allows extraction from common 96-well deep well plates, which enables full or partial automation of the extraction process and yields a large improvement in sample throughput [12].

TFME blades with C_18_ coatings were first used to extract four benzodiazepines from urine and phosphate-buffered saline (PBS) in 2009 [13]. Many other applications of the simultaneous extraction of diverse analytes from complex biological matrices have been reported. Examples include extraction of benzodiazepines from plasma and PBS [14], 49 doping agents from saliva [15], a study of repaglinide metabolism [16], and obtaining fish tissue extracts [17]. C_18_ coatings are also often subjected to comparisons with different coating chemistries in various applications. Selected publications on this subject from the last eight years compare four coatings with regard to the extraction of 110 doping agents from urine [18]; five coatings with regard to the extraction of phenolic compounds from wine, grapes, and berries [19]; seven coatings in metabolomic processes [20]; two coatings with regard to the extraction of abused drugs from plasma, blood, and urine [21]; two coatings with regard to the extraction of beta-blockers and bronchodilators from plasma and urine [11]; and eight chemistries with regard to the systemic evaluation of thin-film microextraction coatings [22]. The results of these studies often demonstrated that C_18_ is the most suitable coating type for a given application.

A common strategy used to increase the efficacy of TFME blades is an implementation of chemistries theoretically more suited for the use with aqueous samples than the conventional C_18_. Most popular solutions include mixed-mode C_18_ (octadecyl with strong cationic exchanger of benzenesulfonic acid) [18,19], polar enhanced (with unspecified weak anionic exchanger) polystyrene-divinylbenzene (PS-DVB) [18,19,20,22,23,24], hydrophilic-lipophilic balance (HLB) [11,20,21,22], and phenylboronic acid (PBA) [18,19,20,22,23] coatings. However, the selection of optimal chemistry is highly dependent on target analytes and their proprieties, with each coating characterised by its unique advantages and limitations. For example, PS-DVB coatings were more efficient than PBA coatings for extraction of diverse small molecules from plasma and PBS. In addition, they did not require preconditioning, unlike PBA coatings [23]. PS-DVB coatings were also superior to C_18_, mixed-mode, or PBA coatings in terms of number of doping agents extracted from urine samples and their amounts, but also were characterized by greater carry-over, what prevented their use for anti-doping control purposes [18]. In another study, divinylbenzene (DVB) coatings were selected over the considered C_18_ coatings for the extraction of six anti-inflammatory and antibacterial drugs from water samples. The reason stated by the authors was DVB’s known suitability for extraction of small molecules from water [25]. Bearing structural similarity to DVB, the HLB particles comprised poly(divinylbenzene-co-N-vinylpyrrolidone) were purposely designed to exert both hydrophobic and hydrophilic type interactions [26] and thus, provide a universal stationary phase for simultaneous extraction of chemically diverse analytes. The benefits of HLB TFME coatings, such as an excellent wettability in aqueous conditions [21], even without preconditioning, were demonstrated for the extraction of nine quaternary ammonium compounds (with mixed hydrophobic-hydrophilic nature) from buffered water [27]. Evaluated by several authors, HLB coatings were superior to the compared alternatives for extraction of small molecules from aqueous media. The examples include better efficacy and lower carry-over than the C_18_ and PS-DVB coatings for the extraction of 25 prohibited substances from plasma [28], greater efficacy than C_18_ for extraction of eight drugs from plasma and urine [11], and greater efficacy than DVB for extraction of six chlorination by-products from hot tub water [26].

However, one fundamental aspect concerning silica-based C_18_ particles has yet to be addressed by researchers. While the stationary phase (coating) is initially wetted in the SPME method during the preconditioning step via insertion into a water–water miscible organic solvent mixture that allows stationary phase wetting to occur under atmospheric pressure [29], the subsequent step of extraction is also performed in a water-based matrix (e.g., plasma, saliva, or urine) under atmospheric pressure. According to the Washburn equation, a pressure of approximately 100 bars (10 MPa) would be required for C_18_-bonded silica particles with 100 Å pores (a commonly used dimension) to remain wet in pure water [30]. Therefore, just as in liquid chromatography, solid phase collapse (dewetting, or chain folding) [1,29] may occur at this stage, potentially altering the efficacy and physicochemical nature of the extraction process.

This study reports the first known attempt to investigate this topic by introducing fully water-compatible C_18_-based stationary phase end-capped with polar groups as a coating of thin-film microextraction blades. A comparison of the performance of these polar and conventional trimethylsilane (TMS) end-capped particles of different sizes is also presented.

## 2. Results and Discussion

### 2.1. Data Quality

This study evaluated four types of SPME coatings and nine different compositions of desorption solvents; in total, 36 unique conditions were tested. Each coating–desorption solvent combination was tested in triplicate. Due to the diverse nature of samples present in different solvents (e.g., unique ionization intensity in electrospray ion source), every result was stacked against the mean value (*n* = 4) that was recorded for the reference sample prepared with a mixture of analytes spiked into the corresponding type of desorption solvent.

Several calibration runs in the expected concentration range of 5–100 ng/mL were performed in all acetonitrile-based, isopropanol-based, and methanol-based desorption solvents. The resulting 7-point calibration curves always provided coefficients of determination exceeding R^2^ = 0.9912 for every analyte with a 1/a^2^ weighting (see Table S1 in Supplementary Materials for more details). Additionally, no problems with carry-over or poor peak shapes were found with any of the analytes, and good reproducibility of the results was achieved. Relative standard deviation (RSD) was below 10% for over 98.6% of the results (median RSD = 3.1%) and did not exceed 15% at any time.

Analysis of the signal recorded for internal standards spiked into desorption solvents confirmed the stability of the detector throughout the experiment. The relative standard deviations in each desorption solvent tested were 4.1–10.1% (median = 5.5%) for oxycodone D_3_, 2.9–8.4% (median = 3.8%) for cocaine D_3_, 8.9–12.5% (median = 10.8%) for alprazolam D_5_, and 3.8–14.9% (median = 5.0%) for THC-COOH D_3_.

### 2.2. Statistical Analysis

Normality was assessed within the dataset for results determined by desorption with the same type of desorption solvent using the Shapiro–Wilk test. The initial results conformed to a normal distribution for every desorption solvent composition except for one, DS3n comprised methanol/water (80/20, *v*/*v*). Following log-transformation, a normal distribution was found in all groups; the testing null hypothesis confirmed the normal distribution of the results. Each group contained an equal number of results, and analysis of variance (ANOVA) performed with the Levene test confirmed the null hypothesis and homoscedasticity of the data. Fulfilment of these conditions allowed us to apply parametrical tests, such as the Pearson correlation coefficient or one-way ANOVA, which are vulnerable to deviations from normality. A one-way ANOVA showed no significant differences in the average values of the dependent variable within the analysed groups.

### 2.3. Quantitative Results (Extraction Efficacy)

With three out of four types of coatings, the desorption solvent labelled as DS3a and composed of methanol/water/formic acid (80/19.9/0.1, *v*/*v*) yielded the best efficacy, except with the 5 µm particles, which also performed worst in this evaluation, where DS1b containing isopropanol/water/ammonium hydroxide (80/19.9/0.1, *v*/*v*) was the most effective. Methanol used as a solvent has been reported to increase steric repulsion of bulky solutes better than acetonitrile [31,32] which may explain the more effective desorption observed with this type of solvent. Steric repulsion is also determined by the density of the ligands bonded with the particle [31]; therefore, the “more crowded” 5 µm particles may have prevented bulky solutes from being intercalated between hydrocarbon chains during the extraction and thus excluded this mechanism from influencing desorption efficacy.

As mentioned before, the poorest extraction efficacy was achieved with coatings prepared with the 5 µm particles. With desorption to the best performing desorption solvent type (DS1b), the extraction efficacy exceeded the median recorded for the entire dataset only in 12 out of 30 analytes (40%), and only a single analyte (methadone) extraction yield was in an upper quarter of the results. With no analyte, the best extraction efficacy was achieved with this type of coating.

With the three remaining coatings, the 10 µm particles yield extraction efficacies above the median for 28 out of 30 analytes (hydrocodone and oxycodone were the exceptions), within the 3rd quartile for 18 analytes (60%); however, the best extraction efficacy was not recorded with this type of coating after desorption to the most effective desorption solvent (DS3a).

The largest tested particles (45 µm) performed much better, and extraction efficacy exceeding the median value was recorded for every single analyte after desorption to DS3a. Additionally, 28 results in the 3rd quartile were recorded, as well as the largest extraction efficacy of the eight analytes.

With Phenomenex^®^ Synergi™ Hydro-RP, which are the only particles end-capped with polar functional groups, results above the median were achieved for all analytes after desorption to DS3a. One less result in the 3rd quartile was recorded than for the 45 µm particles (27 out of 30); however, concurrently, the largest extraction efficacy was recorded for more than double the number of analytes (18 out of 30, or 60%).

**Figure 1:**
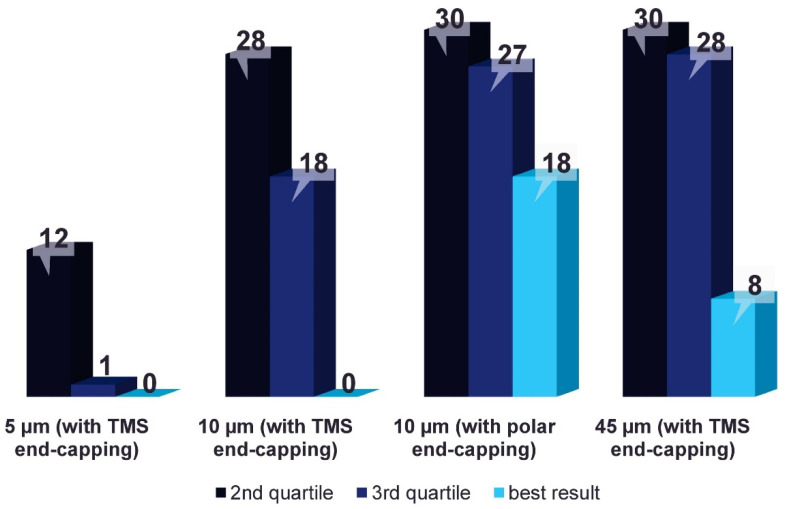
Number of results above the median (2nd quartile), in the 3rd quartile, and the number of best results recorded for the most effective coating–desorption solvent combinations for each coating type. Results for 5 µm particles after desorption to DS1b comprised isopropanol/water/ammonium hydroxide (80/19.9/0.1, *v*/*v*) are shown; results for the other particles after desorption to DS3a comprised methanol/water/formic acid (80/19.9/0.1, *v*/*v*) are shown.

Figure 1 shows the number of results above the median (upper half), in the 3rd quartile (upper quarter) and the number of best results for the most effective coating–desorption solvent combinations for each coating type. The results of statistical analysis for every tested coating–desorption solvent combination are shown in the Supplementary Materials Table S2.

With regard to the top extraction yields for a given analyte, most results (18 = 60% of all) were recorded for the 10 µm particles with polar end-capping groups (all cases after desorption to DS3a) and for the 10 µm particles with TMS end-capping in 2 cases (1 with DS3n and 1 with DS3b). For the 45 µm particles, 10 cases were recorded (8 after desorption to DS3a, 1 with DS1b, and 1 with DS2a). No such results were achieved with the 5 µm particles. The optimal extraction efficacies and optimal conditions for every tested analyte are shown in Table 1, and the comprehensive results with all extraction yields and corresponding relative standard deviations for every analyte are shown in Table S3 in the Supplementary Materials. Median extraction efficacy, extraction efficacy determining the 3rd quartile, as well as top extraction efficacy for given substance are also provided in Table S3 in Supplementary Materials for easier interpretation of the results.

The impact of the physicochemical characteristics of the stationary phases on their extraction performance in this study can be assessed based on the three coatings prepared with TMS end-capped particles. The larger particles performed better than the smaller ones (45 µm > 10 µm > 5 µm). However, this seemed to be an outcome of larger specific surface areas, that were associated with the particles’ sizes (490 m^2^/g > 381 m^2^/g > 338 m^2^/g). In addition, the extraction performance was corelated with the particles’ pore widths. Generally, particles with narrower pores have more crowded ligands [33] (therefore, a greater specific surface area). In this study, pores’ size decreased with an increasing particles’ size (116 Å for 5 µm particles, 104 Å for 10 µm particles, and 64 Å for 45 µm particles), and so increased the particles’ performance. A similar pattern could also be observed for other particle types, such as HLB, where particles with 13 Å pores performed better than particles with 71 Å or 80 Å pores [26]. As noted by the authors, the increased porosity is of an advantage for the extraction of small molecules. With similar pore sizes (71 Å and 80 Å), larger particles performed better for all tested analytes (30–60 µm > 5 µm) [26]. More details on the particles used in this study are shown in Table 2.

### 2.4. Relationship between Extraction Efficacy and Analyte Characteristics

This study evaluated thin-film microextraction devices coated with immobilized octadecyl-bonded silica particles. However, silica surfaces can never be entirely bonded with ligands. Due to steric impedance, it is estimated that below half of the silanol groups present on silica surfaces are bonded with octadecylsilane molecules [1], and part of these silanols becomes permanently inaccessible to end-capping agents such as TMS during synthesis [34,35]. This incomplete substitution of silanols causes multiple interactions between the stationary phase (coating) and analytes to affect the extraction efficacy. This phenomenon also explains why no single parameter of the analyte can be perfectly correlated with extraction yield (i.e., Pearson’s coefficient of exactly r = 1). Several physicochemical properties of the analytes were selected by the authors as potentially affecting extraction, and the correlation of these parameters with the efficacy of the process was investigated. All physicochemical descriptors used for calculations are available in online databases and are shown in Supplementary Materials Table S4.

As expected from a coating prepared with hydrophobic octadecyl-bonded particles, there were two-way significant and strong positive correlations (Pearson’s coefficient values of r > 0.5) between the extraction efficacy and hydrophobicity of the analytes. Expressed as the logarithm of the partition coefficient (logP) or the distribution coefficient (logD), the hydrophobicity of the analytes was computed using several programs. logP values were calculated with the ACD/Labs [36], ALOGPS [37,38], ChemAxon [37,38], and XlogP3.0 [39] programs. logD values at pH 7.4 were calculated with ACD/Labs [36]. Out of this group of descriptors, logD at pH 7.4 was characterized by the highest correlations with extraction efficacy. Given that logD considers whether the molecule is ionizable or not (and majority of the analytes extracted in this study are), these results are not surprising, and the extractions were performed from PBS, which has a nominal pH value of 7.4. Of the four coatings investigated in this study, one prepared with the 45 µm particles exhibits the lowest correlations. The median correlation coefficient value was r = 0.533 (0.495–0.618, *p* ≤ 0.005, *n* = 8). Greater correlations were observed for smaller particle sizes with median value of r = 0.698 (0.615–0.769, *p* < 0.001, *n* = 9) for the 10 µm particles and r = 0.756 (0.612–0.796, *p* < 0.001, *n* = 9) for the 5 µm particles. The largest correlations occurred with the 10 µm particles with polar end-capping with a median value of r = 0.767 (0.646–0.787, *p* < 0.001, *n* = 9). Stronger correlations were also found in the most efficient desorption solvents, achieving maximum values with the 10 and 45 µm particles (both with DS3a) and near-maximum values for the 5 (r = 0.743 in DS1b vs. r = 0.796 in DS1a) and 10 µm particles with polar end-capping (r = 0.784 in DS3a vs. r = 0.787 in DS1b).

Other investigated analyte parameters included the polar surface area [Å^2^], which was computed with ACD/Labs [36], Cactvs 3.4.6.11 [39], and ChemAxon [37,38] programs; the polarizability [Å^3^], which was computed with ACD/Labs [36], and ChemAxon [37,38]; pKa, which was computed with ChemAxon [37,38]; and the number of hydrogen acceptor and donor spots, which were computed with ACD/Labs [36], Cactvs 3.4.6.11 [39] and ChemAxon [37,38]. Of this group of descriptors, two-way significant positive correlations were only observed between the extraction efficacy of the 45 µm particles and the pKa value (strongest acidic) of the analytes. The median coefficient value was r = 0.593 (0.551–0.602, *p* ≤ 0.01, *n* = 5). However, two-way significant negative correlations were present with the extraction yield and pKa value (strongest basic) for the 5 µm particles with a median value of r = −0.488 (−0.545–−0.486, *p* ≤ 0.009, *n* = 3), the 10 µm particles with a median value of r = −0.516 (−0.517–−0.502, *p* ≤ 0.007, *n* = 4), and the 10 µm polar particles with a median value of r = −0.513 (−0.525–−0.497, *p* ≤ 0.007, *n* = 3). Correlations between the extraction efficacy and number of donor spots for hydrogen bonds were also negative for all particle types. Stronger correlations were observed with data from ChemAxon (number of pairs = 28 analytes) than with data from the ACD/Labs and Cactvs programs (*n* = 30 both). With the 5 µm particles, the median correlation value was r = −0.537 (−0.601–−0.480, *p* ≤ 0.01, *n* = 8). With the 10 µm particles, r = −0.544 (−0.643–−0.484, *p* ≤ 0.009, *n* = 9). With the 10 µm polar particles, r = −0.529 (−0.563–−0.493, *p* ≤ 0.008, *n* = 9). With the 45 µm particles, r = −0.611 (−0.698–−0.560, *p* ≤ 0.002, *n* = 9).

All of the two-way significant correlations discussed above are summarised in Table 3.

## 3. Materials and Methods

### 3.1. Particles Used to Prepare TFME Coatings

**Figure 2:**
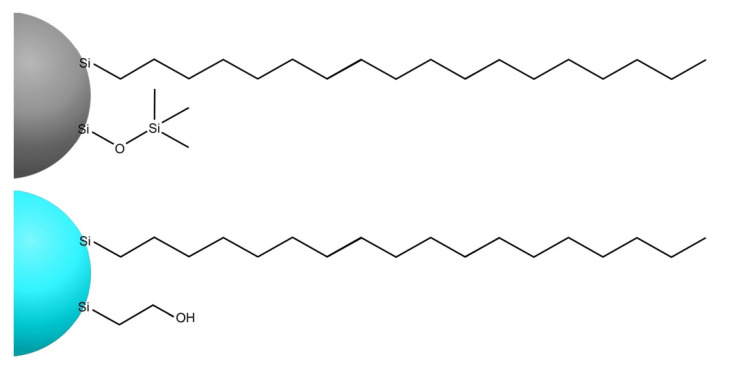
Octadecyl-bonded silica particles with trimethylsilane (**top**) and with polar ethanol (**bottom**) end-capping groups.

In this study, thin-film microextraction coatings were prepared using 4 different types of silica particles bonded to octadecyl ligands and end-capping groups. Macherey Nagel™ NUCLEODUR^®^ C_18_ Htec (henceforth referred to as “5 μm particles”), Phenomenex^®^ Luna^®^ C18(2) (“10 μm particles”), and Macherey-Nagel™ CHROMABOND^®^ C_18_ec (“45 μm particles”) were all end-capped with trimethylsilane, and Phenomenex^®^ Synergi™ Hydro-RP (“10 μm polar end-capped particles”) was end-capped with a polar ethanol group [40]; see Figure 2 for visualization.

Additionally, apart from the differences in end-capping type, important particle parameters included the particles’ size, pore diameter, specific surface area, and total carbon load (see Table 2 for more details).

### 3.2. Preparation of TFME Blades

Thin-film microextraction blades were prepared following a protocol that was previously described by Mirnaghi et al. [10] with certain adjustments including reduction of the drying temperature (from 180 °C to 110 °C) to avoid thermal damage to the coating particles [41].

Pre-cut metal blades (PAS Technology Deutschland GmbH, Magdala, Germany) were etched in concentrated hydrochloric acid (Fluka™, Honeywell International Inc., Charlotte, NC, USA) for 60 min in an ultrasonic bath. The etched blades were cleaned using distilled water and then dried in an oven (150 °C for 30 min).

**Figure 3:**
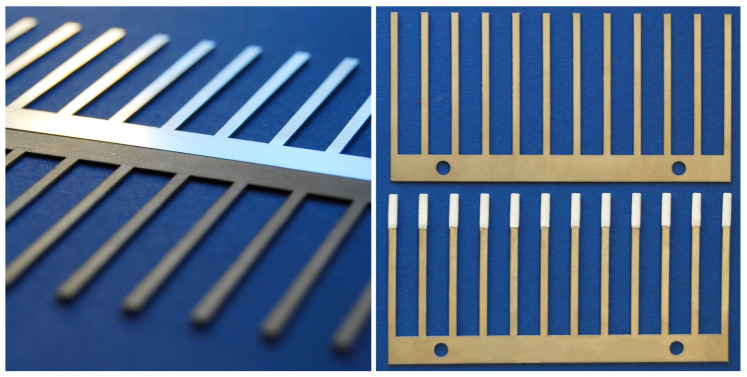
TFME Blades. **Left**: Metal surface before (top) and after (bottom) etching in hydrochloric acid. **Right**: TFME blade before (top) and after (bottom) applying coating.

Next, the blades were covered with 10 layers of previously prepared biocompatible coating with a nitrogen operated sprayer (see Figure 3). After applying each layer, the blades were dried in an oven (110 °C for 3 min). Each type of prepared coating consisted of C_18_-bonded silica particles dispersed in an N,N-dimethylformamide solution of polyacrylonitrile. For every 1.000 g of particles, 7.739 g of N,N-dimethylformamide (Sigma-Aldrich^®^, Merck KGaA, Darmstadt, Germany) and 0.421 g of polyacrylonitrile (Aldrich^®^, Merck KGaA, Darmstadt, Germany) were used.

### 3.3. Reference Standards

Analytical standards of 30 structurally diverse small molecules, such as therapeutic drugs, endogenous hormones, drugs of abuse, doping agents, and their metabolites, were used in this study. The full list presented in alphabetical order includes: 6-acetylcodeine, 11-deoxycortisol, 11-nor-9-carboxy-Δ^9^-tetrahydrocannabinol (THC-COOH), agomelatine, alprazolam, anastrozole, androstenedione, bisoprolol, boldenone, buprenorphine, canrenone, carteolol, clonazepam, cocaine, cortisol, fenoterol, flunitrazepam, hydrocodone, ketamine, lysergic acid diethylamide (LSD), melatonin, methandienone, methadone, methylphenidate, metoprolol, oxycodone, phencyclidine, progesterone, remifentanil acid, and zolpidem.

More details about the reference standards used in this study, including their suppliers, are shown in Supplementary Materials Table S5.

### 3.4. Preparation of the Samples

A testing mixture was prepared by spiking phosphate-buffered saline with stock solutions of all reference substances (to achieve 50 ng/mL concentration). The prepared testing solution was aliquoted to the 96-well DeepWell™ Plates (Nunc™, Thermo Fisher Scientific Inc., Waltham, MA, USA) and used for extractions. A single lot of testing mixture was used throughout the experiment, and all extractions were performed simultaneously to minimize any possible inconsistencies in the results.

A mixture of 4 deuterium-labelled standards (alprazolam D_5_, cocaine D_3_, oxycodone D_3_, and THC-COOH D_3_) was added at a 3 ng/mL concentration to every type of desorption solvent as an internal standard.

### 3.5. Extraction Protocol

All extractions were performed in 96-well plates using plate-compatible SH10 Heater-Shaker (Ingenieurbüro CAT, M. Zipperer GmbH, Ballrechten-Dottingen, Germany). The extraction protocol included: 1st preconditioning (1.5 mL methanol/water (50/50, *v*/*v*), 1 h, 850 rpm agitation); 2nd preconditioning (1 mL methanol/water (50/50, *v*/*v*), 1 h, 850 rpm agitation); 1st rinse (1.5 mL water, 5 s, no agitation); extraction (1 mL of testing mixture (50 ng/mL in PBS), 2.5 h, 850 rpm); 2nd rinse (1.5 mL water, 5 s, no agitation); and desorption (1 mL of desorption solvent, 2 h, 850 rpm). The temperature during the entire protocol was monitored and kept at 18.8 °C. Nine different variants of desorption solvents were used, and their compositions are shown in Table 4. Each variant was spiked with a mixture of deuterium-labelled reference standards at a concentration of 3 ng/mL.

Additionally, the reference samples were transferred to the unoccupied wells of the 96-well plates prior to the desorption step. Therefore, the samples and the reference samples were affected equally by the evaporation of the desorption solvents. Such an approach excluded the impact of the evaporation on the results.

### 3.6. HPLC-MS/MS Method

Samples were analysed with a Shimadzu LCMS-8060 triple quadrupole (Shimadzu Corporation, Kyoto, Japan) system equipped with an Agilent InfinityLab Poroshell 120 EC-C18 analytical column (3 × 100 mm, 2.7 µm) and guard column (3 × 5 mm, 2.7 µm) (Agilent, Santa Clara, CA, USA) using a method previously optimized and described by Sobczak and Goryński [42]. Thus, the column was maintained at 25.0 °C, an injection volume of 0.2 µL was used, and separation was performed in gradient elution mode. Mobile phases consisted of water (LC-MS grade; LiChrosolv^®^, Merck KGaA, Darmstadt, Germany) and acetonitrile (LC-MS grade; CHROMASOLV™, Honeywell International Inc., Charlotte, NC, USA), both with an addition of 0.1% formic acid (LC-MS grade; Optima™, Fisher Chemical, Thermo Fisher Scientific Inc., Waltham, MA, USA). A full list of retention times and monitored precursor–product ion(s) transitions is shown in Table S6 in Supplementary Materials.

### 3.7. Statistical Analysis

The dataset was analysed with IBM SPSS Statistics for Windows, Version 26.0. (IBM Corp, Armonk, NY, USA).

## 4. Conclusions

Thin-film microextraction blades coated with silica particles containing polar end-capping groups provided the best extraction efficacy out of all evaluated types of octadecyl-bonded particles. This type of coating is also characterized by the strongest correlations between the efficacy of the extraction process and hydrophobicity of the analytes, despite not having the highest density of hydrophobic octadecyl ligands. For example, while the hydrophobicity parameter (*H*) of Synergi™ Hydro-RP particles containing polar end-capping groups is higher than the *H* parameter of an average octadecyl particle (according to the hydrophobic-subtraction model [43,44]), the *H* of these particles is lower than the *H* of NUCLEODUR^®^ C_18_ Htec particles [44]. This was not reflected by the results of this study, where the less hydrophobic polar end-capped particles provided better extraction efficacy of the hydrophobic analytes. This suggests that the extraction of small molecules from aqueous samples with the conventional TMS end-capped octadecyl-bonded particles differs from the theoretical expectations. Moreover, in the hydrophobic-subtraction model, polar end-capping groups closely resemble TMS groups [45]; thus, this difference in end-capping type does not provide a satisfactory explanation for the correlations observed with the tested particles. Therefore, one may hypothesize that the extraction yields of TMS end-capped particles are below the theoretical optimal performance due to immersion in a water-based matrix, with which they are not compatible. Such a situation could not occur with water-compatible polar end-capped particles that retained their original characteristics throughout extraction. This reasoning seems to elucidate the benefits of using fully water-compatible stationary phases for extraction from aqueous samples, and was reflected by the results of this study.

Additional investigation is required to assess whether water-compatible polar end-capping can improve extraction efficacy with ligands other than the octadecyl groups that were evaluated in this study. Some examples of stationary phases that are relatively popular in liquid chromatography and susceptible to so-called stationary phase collapse include octyl (C_8_) and phenyl-hexyl. These chemistries could significantly benefit from the incorporation of polar end-capping groups. However, if that were confirmed, then a significant breakthrough in solid phase microextraction could be achieved by preserving all original ligand traits, which may result in improved extraction efficacy. This process may then broaden the range of successfully used liquid chromatography-compatible coatings for future SPME devices and promote methods’ use as a green alternative for sample preparation enabling low organic solvent consumption without compromising the extraction yield.

## Figures and Tables

**Table 1:** Optimal extraction efficacies [%] and conditions. Relative standard deviations [%] given in brackets, *n* = 3, substances arranged by retention order.

Substance	Extraction Efficacy with 10 µm Polar End-Capped Particles and Desorption to DS3a	Optimal Extraction Conditions	
		Extraction Efficacy	Stationary Phase + Desorption Solvent
fenoterol	48.0 (6.7)	65.1 (2.2)	45 µm + DS3a
carteolol	71.0 (3.2)	85.8 (3.0)	45 µm + DS3a
oxycodone	52.4 (8.2)	76.1 (3.5)	45 µm + DS3a
hydrocodone	82.4 (3.4)	93.5 (2.1)	45 µm + DS2a
ketamine	97.8 (1.8)	102.1 (1.9)	45 µm + DS3a
remifentanil acid	63.7 (4.4)	83.9 (1.8)	45 µm + DS3a
metoprolol	90.2 (2.9)	95.1 (3.3)	45 µm + DS3a
6-acetylcodeine	112.5 (2.1)	117.6 (5.7)	10 µm + DS3b
methylphenidate	85.7 (1.4)	94.4 (1.6)	45 µm + DS3a
zolpidem	125.5 (2.5)	10 µm polar end-capped particles + DS3a	
cocaine	102.4 (0.6)	102.8 (0.6)	45 µm + DS1b
LSD	115.0 (4.4)	10 µm polar end-capped particles + DS3a	
melatonin	69.1 (4.8)	89.5 (2.4)	45 µm + DS3a
bisoprolol	120.9 (3.2)	10 µm polar end-capped particles + DS3a	
phencyclidine	114.5 (2.4)	10 µm polar end-capped particles + DS3a	
cortisol	122.9 (5.2)	10 µm polar end-capped particles + DS3a	
buprenorphine	117.0 (4.1)	10 µm polar end-capped particles + DS3a	
alprazolam	120.7 (4.7)	10 µm polar end-capped particles + DS3a	
anastrozole	124.4 (1.6)	10 µm polar end-capped particles + DS3a	
methadone	118.5 (4.3)	10 µm polar end-capped particles + DS3a	
11-deoxycortisol	131.3 (7.4)	10 µm polar end-capped particles + DS3a	
boldenone	131.1 (2.7)	10 µm polar end-capped particles + DS3a	
clonazepam	115.3 (1.3)	10 µm polar end-capped particles + DS3a	
agomelatine	122.8 (3.5)	10 µm polar end-capped particles + DS3a	
methandienone	128.1 (3.0)	10 µm polar end-capped particles + DS3a	
flunitrazepam	124.5 (2.2)	10 µm polar end-capped particles + DS3a	
androstenedione	129.5 (2.9)	10 µm polar end-capped particles + DS3a	
canrenone	131.5 (4.5)	139.9 (1.9)	10 µm + DS3n
progesterone	127.9 (4.8)	10 µm polar end-capped particles + DS3a	
THC-COOH	119.0 (4.3)	10 µm polar end-capped particles + DS3a	

Please note that the extraction efficacies above 100% are due to the evaporation of organic solvents during the desorption step, which occurred under the controlled temperature in open bed configuration of 96-well plates. Desorption solvents compositions: DS1b = isopropanol/water/ammonium hydroxide (80/19.9/0.1, *v*/*v*); DS2a = acetonitrile/water/formic acid (80/19.9/0.1, *v*/*v*); DS3a = methanol/water/formic acid (80/19.9/0.1, *v*/*v*); DS3n = methanol/water (80/20, *v*/*v*); DS3b = methanol/water/ammonium hydroxide (80/19.9/0.1, *v*/*v*).

**Table 2:** Characteristics of particles used to prepare TFME coating.

Particle Type	Nominal Particle Size [μm]	Measured Particle Size [μm]	Pore Diameter [Å]	Specific Surface Area [m^2^/g]	Total Carbon [%]	Surface Coverage [μmole/m^2^]	Compatible pH Range
NUCLEODUR^®^ C_18_ Htec	5	N/A	116	338	18 *	N/A	1–11
Luna^®^ C18(2)	10	8.37	104	381	16.38	3.01	1.5–10
Synergi™ Hydro-RP	10	7.6	88	449	18.9	2.37	1.5–7.5
CHROMABOND^®^ C_18_ec	45	33	64	490	14 *	N/A	2–8

* According to the information provided in product catalogue.

**Table 3:** Two-way significant correlations [Pearson’s r values] discussed in Section 2.4. *p* Values given in brackets.

Parameter	Desorption Solvent	10 µm Polar End-Capped Particles	5 µm Particles	10 µm Particles	45 µm Particles
logD at pH = 7.4 [36]	DS1a	0.780 (0.000)	0.796 (0.000)	0.768 (0.000)	0.576 (0.001)
	DS1n	0.767 (0.000)	0.794 (0.000)	0.752 (0.000)	0.568 (0.001)
	DS1b	0.787 (0.000)	0.743 (0.000)	0.677 (0.000)	0.547 (0.002)
	DS2a	0.782 (0.000)	0.728 (0.000)	0.708 (0.000)	0.513 (0.004)
	DS2n	0.739 (0.000)	0.756 (0.000)	0.698 (0.000)	0.495 (0.005)
	DS2b	0.731 (0.000)	0.763 (0.000)	0.641 (0.000)	0.498 (0.005)
	DS3a	0.784 (0.000)	0.792 (0.000)	0.769 (0.000)	0.618 (0.000)
	DS3n	0.646 (0.000)	0.612 (0.000)	0.615 (0.000)	
	DS3b	0.726 (0.000)	0.683 (0.000)	0.629 (0.000)	0.518 (0.003)
pKa (strongest acidic) [37,38]	DS1a				0.602 (0.004)
	DS1b				0.551 (0.010)
	DS2a				0.593 (0.005)
	DS2b				0.565 (0.008)
	DS3b				0.600 (0.004)
pKa (strongest basic) [37,38]	DS1a		−0.486 (0.009)	−0.515 (0.005)	
	DS2a	−0.525 (0.004)		−0.517 (0.005)	
	DS2n	−0.497 (0.007)	−0.488 (0.008)	−0.502 (0.007)	
	DS3a	−0.513 (0.005)	−0.545 (0.003)	−0.516 (0.005)	
H donors [37,38]	DS1a	−0.493 (0.008)	−0.480 (0.010)	−0.489 (0.008)	−0.590 (0.001)
	DS1n	−0.498 (0.007)	−0.525 (0.004)	−0.531 (0.004)	−0.621 (0.000)
	DS1b	−0.544 (0.003)	−0.585 (0.001)	−0.621 (0.000)	−0.619 (0.000)
	DS2a	−0.526 (0.004)	−0.536 (0.003)	−0.513 (0.005)	−0.611 (0.001)
	DS2n	−0.529 (0.004)	−0.540 (0.003)	−0.556 (0.002)	−0.560 (0.002)
	DS2b	−0.554 (0.002)	−0.535 (0.003)	−0.619 (0.000)	−0.663 (0.000)
	DS3a	−0.494 (0.008)		−0.484 (0.009)	−0.562 (0.002)
	DS3n	−0.559 (0.002)	−0.538 (0.003)	−0.544 (0.003)	−0.584 (0.001)
	DS3b	−0.563 (0.002)	−0.601 (0.001)	−0.643 (0.000)	−0.698 (0.000)

Desorption solvents compositions: DS1a = isopropanol/water/formic acid (80/19.9/0.1, *v*/*v*); DS1n = isopropanol/water (80/20, *v*/*v*); DS1b = isopropanol/water/ammonium hydroxide (80/19.9/0.1, *v*/*v*); DS2a = acetonitrile/water/formic acid (80/19.9/0.1, *v*/*v*); DS2n = acetonitrile/water (80/20, *v*/*v*); DS2b = acetonitrile/water/ammonium hydroxide (80/19.9/0.1, *v*/*v*); DS3a = methanol/water/formic acid (80/19.9/0.1, *v*/*v*); DS3n = methanol/water (80/20, *v*/*v*); DS3b = methanol/water/ammonium hydroxide (80/19.9/0.1, *v*/*v*).

**Table 4:** Composition of desorption solvents.

Desorption Solvent	Composition
DS1a	IPA/W/FA (80/19.9/0.1, *v*/*v*)
DS1n	IPA/W (80/20, *v*/*v*)
DS1b	IPA/W/AH (80/19.9/0.1, *v*/*v*)
DS2a	ACN/W/FA (80/19.9/0.1, *v*/*v*)
DS2n	ACN/W (80/20, *v*/*v*)
DS2b	ACN/W/AH (80/19.9/0.1, *v*/*v*)
DS3a	MeOH/W/FA (80/19.9/0.1, *v*/*v*)
DS3n	MeOH/W (80/20, *v*/*v*)
DS3b	MeOH/W/AH (80/19.9/0.1, *v*/*v*)

Chemicals used: ACN = acetonitrile (LC-MS grade; CHROMASOLV™, Honeywell International Inc., Charlotte, NC, USA); AH = ammonium hydroxide (LC-MS grade; Fluka™, Honeywell International Inc., Charlotte, NC, USA); FA = formic acid (LC-MS grade; Optima™, Fisher Chemical, Thermo Fisher Scientific Inc., Waltham, MA, USA); IPA = isopropanol (LC-MS grade; CHROMASOLV™, Honeywell International Inc., Charlotte, NC, USA); MeOH = methanol (LC-MS grade; CHROMASOLV™, Honeywell International Inc., Charlotte, NC, USA, W = water (LC-MS grade; LiChrosolv^®^, Merck KGaA, Darmstadt, Germany).

## Data Availability

The data presented in this study are available on request from the corresponding author.

## Supplementary Materials

The following are available online, Table S1. Coefficients of determination (R^2^) determined during calibration runs in different desorption solvents.; Table S2. Number of results in each category for every stationary phase—desorption solvent combination.; Table S3. Extraction efficacies [%] for each stationary phase—desorption solvent combination.; Table S4. Essential physicochemical proprieties of the analysed substances.; Table S5. List of reference standards in alphabetical order.; Table S6. Monitored precursor—product ion(s) transitions.
