# Supplementary material for: Benefits of Innovative and Fully Water-Compatible Stationary Phases of Thin-Film Microextraction (TFME) Blades
Source: Molecules. 2021 Jul 21;26(15):4413. doi: 10.3390/molecules26154413 (PMC8347298; doi:10.3390/molecules26154413)
Supplement: Supplementary file 1 [file molecules-26-04413-s001.zip › molecules-1286508-supplementary.pdf]

Supplementary Material for:

Benefits of innovative and fully water-compatible stationary phases of thin-film microextraction (TFME) blades

by Łukasz Sobczak, Dominika Kołodziej and Krzysztof Goryński

List of contents:

Table S1. Coefficients of determination (R^2) determined during calibration runs in different desorption solvents.

Table S2. Number of results in each category for every stationary phase – desorption solvent combination.

Table S3. Extraction efficacies [%] for each stationary phase – desorption solvent combination.

Table S4. Essential physicochemical proprieties of the analysed substances.

Table S5. List of reference standards in alphabetical order.

Table S6. Monitored precursor – product ion(s) transitions.

Table S1. Coefficients of determination (R^2) determined during calibration runs in different desorption solvents. *Substances arranged by retention order.*

substance	coefficient of determination (R^2) in desorption solvent composition:		
	isopropanol/water (80/20, v/v)	acetonitrile/water (80/20, v/v)	methanol/water (80/20, v/v)
fenoterol	0.9996	0.9999	0.9982
carteolol	0.9993	0.9996	1.0000
oxycodone	0.9991	0.9985	0.9984
hydrocodone	0.9992	0.9998	0.9991
ketamine	0.9989	0.9998	1.0000
remifentanil acid	0.9993	0.9984	0.9995
metoprolol	1.0000	0.9998	0.9980
6-acetylcodeine	0.9995	0.9998	1.0000
methylphenidate	0.9999	0.9998	0.9995
zolpidem	1.0000	0.9991	1.0000
cocaine	0.9994	0.9999	0.9989
LSD	0.9999	0.9999	0.9973
melatonin	0.9999	0.9997	0.9996
bisoprolol	0.9998	0.9999	0.9997
phencyclidine	0.9998	0.9993	0.9991
cortisol	0.9978	0.9994	0.9998
buprenorphine	0.9996	0.9992	0.9999
alprazolam	0.9975	0.9997	0.9989
anastrozole	1.0000	0.9997	0.9984
methadone	0.9982	1.0000	0.9999
11-deoxycortisol	0.9944	0.9999	0.9978
boldenone	1.0000	0.9981	0.9988
clonazepam	0.9991	0.9976	0.9991
agomelatine	0.9989	0.9998	0.9999
methandienone	0.9993	0.9999	0.9980
flunitrazepam	0.9998	0.9999	0.9999
androstenedione	0.9990	0.9991	0.9999
canrenone	0.9919	0.9912	0.9999
progesterone	0.9999	0.9998	0.9999
THC-COOH	0.9978	0.9998	1.0000

Table S2. Number of results in each category for every stationary phase – desorption solvent combination. *Table arranged by the type of stationary phase.*

stationary phase + desorption solvent combination	number of results:		
	above median (in Q_2)	in 3rd quartile (Q_3)	best results
5 µm + DS1a	2	-	-
5 µm + DS1n	-	-	-
5 µm + DS1b	12	1	-
5 µm + DS2a	5	-	-
5 µm + DS2n	-	-	-
5 µm + DS2b	6	-	-
5 µm + DS3a	-	-	-
5 µm + DS3n	1	1	-
5 µm + DS3b	10	1	-
10 µm + DS1a	22	17	-
10 µm + DS1n	6	1	-
10 µm + DS1b	28	6	-
10 µm + DS2a	29	13	-
10 µm + DS2n	28	13	-
10 µm + DS2b	23	1	-
10 µm + DS3a	28	18	-
10 µm + DS3n	14	5	1
10 µm + DS3b	28	8	1
10 µm with polar end-capping + DS1a	10	2	-
10 µm with polar end-capping + DS1n	3	-	-
10 µm with polar end-capping + DS1b	9	5	-
10 µm with polar end-capping + DS2a	20	15	-
10 µm with polar end-capping + DS2n	20	8	-
10 µm with polar end-capping + DS2b	-	-	-
10 µm with polar end-capping + DS3a	30	27	18
10 µm with polar end-capping + DS3n	5	1	-
10 µm with polar end-capping + DS3b	3	2	-
45 µm + DS1a	24	14	-
45 µm + DS1n	14	4	-
45 µm + DS1b	29	26	1
45 µm + DS2a	25	12	1
45 µm + DS2n	23	12	-
45 µm + DS2b	18	10	-
45 µm + DS3a	30	28	8
45 µm + DS3n	15	7	-
45 µm + DS3b	20	12	-

Desorption solvents compositions: DS1a = isopropanol/water/formic acid (80/19.9/0.1, v/v); DS1n = isopropanol/water (80/20, v/v); DS1b = isopropanol/water/ammonium hydroxide (80/19.9/0.1, v/v); DS2a = acetonitrile/water/formic acid (80/19.9/0.1, v/v); DS2n = acetonitrile/water (80/20, v/v); DS2b = acetonitrile/water/ammonium hydroxide (80/19.9/0.1, v/v); DS3a = methanol/water/formic acid (80/19.9/0.1, v/v); DS3n = methanol/water (80/20, v/v); DS3b = methanol/water/ammonium hydroxide (80/19.9/0.1, v/v).

Table S3. Extraction efficacies [%] for each stationary phase – desorption solvent combination (SP + DS). *Relative standard deviations [%] given in brackets, n=3, substances arranged by retention order.*

SP + DS ↓ / → substance	fenoterol	carteolol	oxycodone	hydrocodone	ketamine	remifentanil acid	metoprolol	6-acetylcodeine	methylphenidate	zolpidem	cocaine	LSD	melatonin	bisoprolol	phencyclidine	cortisol	buprenorphine	alprazolam	anastrozole	methadone	11-deoxycortisol	boldenone	clonazepam	agomelatine	methandienone	flunitrazepam	androstenedione	canrenone	progesterone	THC-COOH
**median extraction efficacy**	**35.3**	**61.2**	**48.1**	**68.8**	**79.0**	**59.3**	**75.5**	**99.6**	**69.3**	**102.5**	**85.5**	**97.7**	**56.7**	**97.8**	**89.0**	**96.4**	**91.6**	**99.9**	**97.7**	**96.7**	**101.9**	**102.3**	**92.3**	**96.5**	**100.8**	**96.9**	**103.4**	**104.4**	**98.6**	**79.5**
**3rd quartile extraction efficacy**	**45.2**	**71.2**	**60.3**	**81.6**	**87.0**	**70.3**	**84.9**	**104.5**	**80.3**	**105.8**	**94.2**	**101.1**	**66.9**	**102.6**	**94.5**	**101.4**	**95.2**	**104.5**	**104.1**	**101.3**	**106.3**	**107.9**	**99.4**	**104.2**	**106.4**	**103.8**	**106.8**	**111.9**	**103.0**	**86.6**
**top extraction efficacy**	**65.1**	**85.8**	**76.1**	**93.5**	**102.1**	**83.9**	**95.1**	**117.6**	**94.4**	**125.5**	**102.8**	**115.0**	**89.5**	**120.9**	**114.5**	**122.9**	**117.0**	**120.7**	**124.4**	**118.5**	**131.3**	**131.1**	**115.3**	**122.8**	**128.1**	**124.5**	**129.5**	**139.9**	**127.9**	**119.0**
10 µm + DS1a	38.9 (8.3)	60.2 (6.8)	47.8 (7.7)	65.6 (6.6)	75.0 (4.1)	59.8 (8.2)	79.8 (3.5)	95.5 (2.1)	68.5 (3.9)	106.2 (2.3)	83.7 (2.9)	93.8 (2.6)	57.6 (6.7)	104.0 (0.9)	96.8 (1.6)	105.4 (2.7)	100.0 (2.6)	103.9 (2.4)	104.8 (1.8)	106.9 (2.0)	110.7 (1.3)	109.7 (0.6)	101.7 (1.5)	104.7 (1.0)	113.0 (1.8)	106.8 (1.9)	111.6 (2.4)	113.7 (3.1)	111.7 (2.1)	97.7 (5.4)
10 µm polar + DS1a	35.7 (8.1)	55.6 (7.4)	40.4 (3.1)	66.5 (5.0)	73.5 (4.2)	46.3 (4.9)	71.2 (9.0)	89.2 (4.6)	65.3 (7.1)	101.7 (3.4)	78.1 (5.6)	92.9 (4.4)	50.9 (2.4)	95.4 (4.1)	90.5 (4.9)	97.2 (5.1)	94.0 (2.7)	96.4 (5.4)	94.7 (3.6)	100.2 (3.5)	106.6 (1.9)	102.2 (4.9)	87.5 (5.6)	94.3 (3.7)	101.3 (4.2)	93.5 (3.1)	102.5 (3.3)	105.0 (2.7)	102.2 (2.6)	91.0 (1.9)
5 µm + DS1a	25.5 (5.3)	40.0 (6.9)	29.4 (10.4)	45.5 (5.3)	59.4 (2.9)	36.4 (6.0)	54.0 (2.2)	82.4 (1.1)	48.3 (4.9)	99.2 (3.2)	67.0 (1.7)	88.1 (2.0)	43.7 (3.2)	89.5 (2.6)	82.0 (2.8)	92.3 (3.8)	90.0 (8.7)	93.7 (3.3)	87.2 (4.6)	99.3 (3.3)	97.3 (4.0)	97.9 (1.3)	83.1 (3.8)	90.5 (3.8)	96.6 (1.5)	90.5 (2.9)	104.3 (2.5)	101.5 (3.8)	97.1 (9.6)	76.0 (9.1)
45 µm + DS1a	60.1 (6.8)	79.5 (6.3)	72.4 (6.5)	90.2 (3.3)	94.3 (3.5)	77.4 (5.3)	89.5 (7.9)	99.1 (3.4)	87.8 (6.5)	102.8 (3.4)	90.3 (4.6)	100.5 (3.1)	83.5 (6.2)	102.8 (5.2)	99.2 (4.6)	106.1 (3.2)	87.9 (4.4)	104.1 (2.7)	103.8 (5.9)	101.1 (3.1)	101.0 (4.0)	105.1 (2.8)	103.8 (2.8)	104.1 (3.4)	105.1 (1.7)	104.2 (3.6)	105.8 (1.7)	103.9 (2.3)	95.8 (2.5)	71.4 (2.4)
10 µm + DS1n	30.8 (8.1)	54.5 (3.7)	41.3 (6.6)	61.2 (1.9)	72.9 (2.6)	53.6 (4.5)	68.7 (4.0)	91.9 (1.8)	63.7 (2.0)	100.4 (0.5)	83.9 (2.8)	83.6 (2.9)	44.8 (2.3)	94.6 (4.5)	89.5 (4.9)	93.2 (3.1)	95.3 (1.5)	98.6 (2.9)	94.2 (1.0)	100.6 (5.9)	104.9 (6.7)	100.6 (2.2)	92.0 (1.8)	94.6 (2.0)	99.0 (2.3)	96.6 (1.7)	101.8 (3.1)	100.0 (3.1)	100.4 (2.1)	85.9 (2.9)
10 µm polar + DS1n	28.5 (6.4)	49.7 (1.3)	33.3 (5.0)	58.7 (4.7)	70.3 (3.1)	43.6 (7.0)	62.1 (4.1)	86.7 (2.4)	59.0 (3.0)	98.1 (0.9)	75.6 (1.9)	83.0 (4.8)	41.0 (7.6)	90.3 (0.7)	82.7 (2.7)	90.5 (1.6)	93.5 (1.5)	94.0 (0.9)	88.4 (3.9)	96.4 (5.3)	103.3 (7.5)	97.4 (0.8)	84.9 (3.7)	90.8 (0.8)	96.6 (1.9)	90.6 (4.1)	98.5 (3.4)	97.5 (2.4)	95.8 (1.4)	80.6 (5.2)
5 µm + DS1n	23.1 (3.1)	39.5 (1.7)	29.3 (2.3)	43.9 (5.2)	59.4 (1.8)	34.3 (6.1)	52.4 (2.6)	77.4 (0.8)	47.6 (3.4)	92.2 (2.2)	67.6 (2.0)	75.1 (5.8)	35.1 (6.8)	80.5 (2.5)	77.7 (4.0)	80.1 (1.8)	84.6 (1.4)	85.5 (3.6)	78.8 (3.4)	92.3 (5.0)	86.0 (3.4)	88.2 (1.3)	73.7 (1.2)	81.9 (0.9)	86.8 (3.0)	83.1 (1.6)	93.9 (1.8)	95.1 (1.4)	89.5 (1.8)	73.8 (5.2)
45 µm + DS1n	47.8 (1.6)	71.7 (3.7)	59.5 (4.4)	81.4 (2.3)	88.7 (1.6)	69.0 (0.9)	80.9 (3.7)	95.6 (2.2)	80.9 (3.2)	99.7 (1.2)	91.1 (2.5)	88.7 (0.2)	63.7 (1.9)	96.7 (3.1)	93.1 (4.7)	97.2 (1.3)	81.6 (4.8)	98.1 (1.3)	94.7 (1.1)	97.2 (3.9)	94.7 (4.6)	98.1 (0.7)	94.6 (0.5)	94.9 (1.2)	98.6 (1.7)	96.2 (2.2)	97.8 (1.7)	95.4 (2.3)	88.3 (3.8)	67.1 (2.3)
10 µm + DS1b	39.8 (12.8)	70.4 (8.2)	60.8 (10.1)	76.0 (6.2)	84.6 (4.8)	68.1 (8.4)	84.9 (7.2)	99.6 (3.3)	79.8 (6.5)	104.0 (4.0)	95.0 (2.8)	95.1 (4.3)	59.6 (10.6)	103.5 (1.6)	103.4 (3.6)	101.3 (4.9)	98.2 (2.3)	104.4 (3.2)	101.4 (5.1)	109.2 (2.9)	102.9 (6.2)	104.9 (3.7)	98.2 (5.3)	99.7 (4.7)	100.9 (4.6)	103.5 (3.8)	106.6 (2.7)	105.8 (2.7)	101.8 (1.4)	80.0 (3.6)
10 µm polar + DS1b	27.2 (5.4)	51.8 (9.5)	39.7 (7.9)	62.4 (2.7)	77.4 (5.4)	44.4 (5.6)	66.1 (3.4)	94.9 (0.5)	65.1 (2.5)	103.0 (1.1)	84.7 (0.4)	94.4 (1.2)	44.5 (4.4)	97.7 (1.8)	96.3 (2.3)	96.1 (4.7)	103.1 (6.0)	100.8 (5.1)	94.9 (0.7)	106.7 (4.0)	100.1 (8.2)	104.8 (0.1)	87.6 (2.4)	92.4 (1.4)	100.1 (1.7)	94.4 (2.3)	103.3 (2.3)	105.3 (5.0)	105.5 (3.4)	92.9 (13.7)
5 µm + DS1b	34.0 (14.1)	61.0 (9.4)	51.1 (11.4)	65.9 (9.0)	79.0 (5.5)	55.9 (8.7)	74.8 (10.7)	94.0 (2.9)	71.0 (9.0)	100.2 (1.8)	89.3 (4.5)	93.3 (1.9)	55.9 (9.6)	96.8 (3.1)	94.2 (4.2)	95.7 (4.2)	95.2 (2.5)	97.4 (1.7)	95.5 (1.6)	105.1 (4.3)	103.2 (2.0)	100.2 (1.1)	90.8 (1.6)	97.5 (1.4)	101.5 (2.5)	97.1 (3.8)	105.3 (2.1)	103.0 (1.9)	100.8 (2.5)	77.0 (3.0)
45 µm + DS1b	53.5 (2.5)	84.0 (2.7)	76.1 (2.3)	92.0 (0.9)	98.9 (1.1)	81.2 (0.5)	90.4 (3.2)	105.9 (2.3)	94.3 (2.2)	106.8 (1.2)	102.8 (0.6)	101.9 (1.7)	77.1 (0.6)	107.8 (2.8)	105.8 (3.4)	110.2 (1.4)	92.7 (3.0)	108.3 (3.3)	106.0 (1.7)	107.3 (5.7)	113.0 (2.3)	110.3 (5.4)	101.8 (2.7)	109.5 (1.3)	108.3 (1.1)	109.3 (2.5)	107.9 (0.9)	107.2 (6.4)	98.6 (1.7)	74.8 (3.0)
10 µm + DS2a	44.8 (6.2)	69.2 (1.5)	56.6 (3.9)	75.1 (2.2)	81.9 (1.9)	69.5 (4.7)	84.2 (3.4)	100.2 (0.5)	74.0 (1.8)	105.0 (2.6)	92.0 (1.0)	98.5 (1.0)	64.0 (4.5)	103.4 (1.0)	92.8 (1.2)	106.3 (1.8)	97.1 (2.9)	103.2 (1.4)	106.4 (2.9)	96.4 (1.8)	110.9 (3.1)	110.0 (4.4)	102.3 (2.1)	107.0 (1.1)	110.1 (1.3)	107.8 (2.0)	114.4 (1.5)	111.3 (1.3)	106.4 (2.2)	89.7 (1.1)
10 µm polar + DS2a	33.9 (6.1)	52.5 (3.6)	40.5 (5.5)	67.4 (3.3)	80.8 (1.9)	46.9 (2.0)	70.9 (5.6)	99.4 (0.7)	66.7 (3.8)	108.0 (2.0)	85.2 (1.9)	101.2 (1.4)	52.2 (3.4)	102.3 (1.2)	93.0 (2.2)	102.0 (5.6)	96.2 (3.3)	105.3 (3.5)	105.0 (1.3)	99.8 (0.7)	110.3 (1.2)	112.1 (1.5)	97.7 (1.1)	105.6 (1.0)	113.0 (0.6)	105.1 (2.3)	116.5 (1.8)	115.5 (1.1)	111.5 (1.9)	96.3 (2.6)
5 µm + DS2a	42.4 (10.5)	61.4 (8.5)	48.4 (7.1)	67.2 (5.9)	74.2 (5.2)	58.7 (7.5)	75.4 (5.5)	90.4 (3.1)	66.5 (8.0)	98.5 (1.7)	83.2 (4.7)	91.5 (3.1)	58.9 (7.5)	95.7 (3.6)	87.7 (4.5)	91.8 (3.7)	87.2 (1.1)	93.3 (1.1)	96.3 (3.0)	94.2 (1.7)	103.0 (4.4)	99.2 (5.0)	88.9 (4.0)	94.0 (3.7)	98.4 (2.2)	95.9 (2.4)	103.3 (2.9)	101.9 (3.8)	98.3 (0.1)	78.4 (1.3)
45 µm + DS2a	59.2 (3.8)	80.4 (3.5)	71.4 (3.6)	93.5 (2.1)	93.3 (1.0)	79.8 (2.8)	91.1 (2.5)	101.6 (2.3)	86.0 (2.9)	103.6 (2.6)	93.0 (2.0)	99.8 (2.3)	78.5 (5.2)	102.6 (2.2)	94.0 (1.4)	100.8 (2.1)	86.2 (1.8)	103.3 (6.0)	107.4 (3.0)	95.3 (4.0)	101.6 (5.8)	107.3 (5.0)	103.5 (1.8)	104.0 (1.9)	103.9 (2.1)	104.8 (2.0)	104.9 (2.4)	105.6 (4.3)	94.5 (3.4)	71.8 (3.3)
10 µm + DS2n	40.2 (7.8)	65.0 (3.0)	57.7 (4.4)	77.8 (2.7)	85.8 (1.9)	70.2 (4.8)	81.7 (4.6)	102.6 (1.9)	76.4 (3.6)	109.2 (1.6)	94.6 (4.4)	106.8 (1.7)	66.3 (3.9)	99.4 (2.4)	85.6 (4.8)	103.3 (3.7)	94.7 (0.4)	107.9 (2.2)	105.4 (2.3)	86.8 (6.0)	105.6 (3.0)	108.6 (1.4)	100.8 (2.5)	108.2 (0.7)	109.7 (2.2)	103.6 (1.8)	107.6 (3.0)	107.4 (2.0)	106.9 (3.1)	89.3 (3.6)
10 µm polar + DS2n	32.6 (5.3)	53.7 (7.7)	41.3 (5.6)	69.1 (2.7)	82.1 (0.8)	51.1 (0.9)	73.4 (6.5)	99.7 (1.5)	67.8 (6.7)	108.6 (2.8)	85.9 (3.7)	105.5 (2.5)	52.3 (2.3)	97.9 (4.7)	81.9 (4.2)	99.2 (5.0)	94.7 (1.7)	106.3 (3.3)	101.3 (2.9)	87.7 (5.0)	106.5 (2.4)	107.7 (1.6)	91.7 (0.9)	105.5 (2.2)	108.9 (2.6)	101.5 (1.8)	108.7 (2.2)	110.5 (1.4)	108.9 (0.8)	86.3 (1.7)
5 µm + DS2n	28.6 (3.5)	49.5 (5.6)	38.1 (8.3)	58.0 (5.9)	68.1 (4.5)	47.2 (6.1)	61.3 (8.5)	87.0 (3.2)	56.3 (5.7)	97.9 (4.7)	77.7 (6.9)	90.8 (5.1)	47.4 (5.3)	87.8 (7.5)	77.6 (6.5)	87.8 (4.8)	82.4 (4.4)	94.3 (3.9)	90.4 (5.4)	84.6 (8.2)	94.8 (4.0)	95.2 (5.5)	80.1 (5.0)	91.4 (6.1)	97.3 (5.0)	91.1 (5.2)	101.0 (3.0)	94.7 (6.1)	92.1 (4.1)	77.6 (3.3)
45 µm + DS2n	53.3 (5.7)	76.9 (3.6)	64.9 (1.9)	90.0 (0.8)	93.4 (1.6)	79.1 (1.3)	85.1 (1.5)	104.1 (0.7)	85.8 (1.1)	105.6 (0.7)	96.5 (2.2)	104.1 (1.3)	77.3 (1.2)	96.6 (2.7)	81.4 (3.0)	98.2 (3.2)	86.7 (1.4)	105.9 (3.0)	102.3 (3.1)	81.6 (2.6)	103.7 (4.5)	102.5 (2.9)	99.0 (2.2)	101.6 (3.3)	105.8 (3.2)	100.7 (1.8)	105.5 (2.8)	100.0 (3.5)	93.6 (0.7)	71.2 (4.6)

SP + DS ↓ / substance →	fenoterol	carteolol	oxycodone	hydrocodone	ketamine	remifentanil acid	metoprolol	6-acetylcodeine	methylphenidate	zolpidem	cocaine	LSD	melatonin	bisoprolol	phencyclidine	cortisol	buprenorphine	alprazolam	anastrozole	methadone	11-deoxycortisol	boldenone	clonazepam	agomelatine	methandienone	flunitrazepam	androstenedione	canrenone	progesterone	THC-COOH
10 µm + DS2b	29.6 (10.9)	68.2 (5.5)	57.7 (7.3)	74.6 (6.4)	84.8 (5.3)	68.4 (8.3)	84.2 (5.5)	100.0 (3.9)	76.9 (6.3)	102.2 (3.7)	94.1 (5.6)	101.1 (3.3)	62.8 (6.8)	102.7 (4.5)	92.9 (3.9)	97.6 (5.5)	92.2 (5.5)	102.6 (3.6)	99.2 (4.6)	97.0 (3.8)	102.2 (4.6)	104.5 (4.1)	96.3 (4.4)	97.7 (4.6)	99.5 (5.2)	101.0 (4.9)	102.6 (5.2)	102.0 (3.0)	97.0 (3.8)	79.1 (2.3)
10 µm polar + DS2b	21.9 (3.0)	52.0 (5.1)	37.8 (4.1)	62.2 (2.5)	73.7 (4.0)	46.0 (2.7)	69.3 (5.2)	88.3 (3.0)	62.0 (4.7)	97.2 (3.7)	81.6 (5.5)	94.7 (3.0)	48.0 (4.5)	93.2 (3.5)	82.3 (4.3)	94.1 (4.5)	83.5 (5.4)	94.4 (1.9)	89.9 (3.4)	90.8 (5.0)	95.8 (1.7)	99.3 (5.5)	82.4 (2.1)	95.2 (4.2)	98.0 (1.8)	91.1 (2.6)	96.0 (2.7)	96.1 (4.4)	96.3 (7.9)	77.0 (6.5)
5 µm + DS2b	23.9 (10.9)	51.7 (5.6)	39.6 (4.0)	58.3 (5.8)	72.2 (3.4)	48.5 (8.0)	68.4 (6.3)	90.5 (1.6)	59.5 (4.0)	100.1 (2.2)	82.3 (1.2)	97.2 (2.0)	51.4 (6.9)	93.7 (2.2)	86.0 (1.5)	95.0 (0.8)	90.9 (4.5)	98.3 (1.6)	93.6 (0.4)	98.7 (2.8)	106.2 (6.9)	100.8 (3.2)	87.5 (1.4)	95.5 (0.9)	101.0 (2.0)	93.7 (1.1)	103.4 (1.1)	102.5 (1.0)	98.8 (3.2)	79.8 (2.0)
45 µm + DS2b	35.0 (6.5)	76.0 (8.1)	68.3 (8.0)	87.1 (4.0)	93.5 (2.8)	77.0 (4.0)	88.7 (3.1)	99.9 (1.6)	84.9 (5.1)	100.8 (1.9)	96.0 (3.5)	102.2 (0.6)	75.7 (5.7)	98.5 (3.9)	86.8 (4.2)	96.8 (0.5)	83.6 (1.3)	101.5 (1.1)	100.7 (2.0)	89.3 (3.0)	98.3 (0.9)	101.4 (2.8)	97.3 (2.7)	101.5 (0.4)	100.6 (2.2)	99.7 (1.2)	100.8 (1.9)	98.1 (4.3)	91.9 (2.8)	70.3 (4.3)
10 µm + DS3a	40.5 (9.1)	63.6 (8.5)	47.0 (8.8)	68.5 (5.7)	79.1 (5.7)	61.5 (8.8)	82.5 (6.5)	100.8 (5.3)	71.7 (7.4)	113.7 (3.1)	91.2 (4.4)	100.9 (2.1)	59.1 (9.6)	109.4 (3.6)	103.7 (3.9)	112.8 (1.4)	105.9 (2.6)	109.8 (6.1)	112.3 (4.2)	110.0 (4.6)	116.2 (2.5)	117.5 (4.4)	105.7 (3.5)	113.5 (4.2)	115.7 (2.3)	113.0 (3.2)	119.9 (2.4)	120.3 (1.4)	116.2 (0.7)	106.4 (0.9)
10 µm polar + DS3a	48.0 (6.7)	71.0 (3.2)	52.4 (8.2)	82.4 (3.4)	97.8 (1.8)	63.7 (4.4)	90.2 (2.9)	112.5 (2.1)	85.7 (1.4)	125.5 (2.5)	102.4 (0.6)	115.0 (4.4)	69.1 (4.8)	120.9 (3.2)	114.5 (2.4)	122.9 (5.2)	117.0 (4.1)	120.7 (4.7)	124.4 (1.6)	118.5 (4.3)	131.3 (7.4)	131.1 (2.7)	115.3 (1.3)	122.8 (3.5)	128.1 (3.0)	124.5 (2.2)	129.5 (2.9)	131.5 (4.5)	127.9 (4.8)	119.0 (4.3)
5 µm + DS3a	32.4 (8.5)	49.4 (4.6)	36.3 (5.4)	50.3 (6.6)	67.8 (4.9)	45.4 (9.1)	62.1 (3.8)	79.9 (1.3)	55.6 (6.3)	95.3 (1.5)	70.5 (3.8)	82.2 (2.3)	50.9 (6.8)	89.5 (2.6)	82.3 (2.6)	91.0 (2.2)	86.6 (3.4)	90.3 (4.8)	92.5 (2.6)	90.0 (0.8)	101.1 (2.7)	96.9 (2.4)	85.9 (3.9)	93.5 (3.2)	97.8 (1.0)	92.6 (3.5)	100.2 (1.9)	102.8 (5.4)	98.5 (4.4)	78.9 (6.9)
45 µm + DS3a	65.1 (2.2)	85.8 (3.0)	76.1 (3.5)	92.3 (2.6)	102.1 (1.9)	83.9 (1.8)	95.1 (3.3)	105.7 (0.2)	94.4 (1.6)	109.9 (0.6)	100.0 (1.3)	105.5 (0.7)	89.5 (2.4)	108.6 (0.7)	104.7 (1.3)	110.7 (2.3)	92.2 (2.4)	105.1 (0.8)	116.7 (1.5)	103.4 (1.3)	119.1 (0.4)	114.0 (2.2)	108.9 (1.8)	113.3 (1.7)	113.1 (0.3)	112.4 (0.4)	114.4 (2.7)	114.0 (3.8)	107.8 (2.8)	83.2 (9.0)
10 µm + DS3n	35.6 (12.1)	52.8 (9.6)	41.0 (11.3)	69.1 (8.1)	71.6 (6.4)	62.0 (10.9)	64.9 (4.3)	108.2 (1.7)	55.4 (8.4)	107.4 (2.4)	71.2 (5.3)	100.3 (1.4)	38.8 (12.3)	98.2 (2.1)	78.0 (2.1)	74.7 (1.6)	95.5 (3.6)	99.0 (2.7)	99.4 (3.4)	90.9 (6.1)	85.0 (2.8)	108.7 (2.7)	90.5 (3.4)	76.8 (2.1)	94.6 (3.5)	86.0 (3.7)	103.8 (2.6)	139.9 (1.9)	99.9 (2.1)	84.0 (4.3)
10 µm polar + DS3n	29.6 (6.2)	43.9 (2.3)	32.6 (6.4)	65.8 (4.3)	68.5 (6.1)	45.8 (8.7)	56.9 (3.5)	102.9 (3.5)	53.1 (5.2)	105.4 (1.8)	66.2 (2.1)	99.6 (2.9)	32.6 (6.2)	94.7 (1.2)	75.1 (1.4)	69.9 (3.8)	89.2 (4.1)	93.8 (3.8)	90.2 (3.8)	91.7 (7.9)	83.6 (5.7)	102.4 (0.8)	83.0 (3.7)	71.8 (2.5)	91.7 (2.7)	81.0 (2.1)	100.0 (1.9)	133.7 (4.1)	95.7 (2.8)	76.0 (2.4)
5 µm + DS3n	32.2 (3.7)	43.8 (5.0)	34.1 (3.6)	57.8 (1.8)	60.6 (0.8)	49.6 (3.0)	54.9 (4.3)	87.9 (1.5)	47.4 (4.3)	89.4 (1.3)	58.9 (3.7)	86.6 (2.8)	34.7 (1.7)	82.0 (4.1)	63.3 (5.8)	62.4 (2.3)	77.0 (3.3)	79.5 (1.1)	81.5 (1.5)	76. 4 (6.9)	71.2 (2.7)	89.3 (2.6)	73.6 (3.7)	63.2 (2.1)	78.0 (3.0)	72.2 (1.2)	87.8 (2.2)	117.8 (2.6)	85.7 (1.9)	68.4 (2.2)
45 µm + DS3n	54.9 (1.5)	68.2 (4.2)	61.2 (4.5)	92.3 (3.7)	82.3 (3.2)	79.8 (3.1)	75.6 (4.5)	110.5 (3.8)	70.1 (2.2)	103.8 (2.4)	75.9 (2.6)	102.4 (3.1)	54.5 (2.2)	97.9 (2.1)	78.5 (2.1)	74.0 (4.1)	83.5 (2.2)	93.2 (3.0)	97.3 (2.9)	90.1 (4.1)	84.3 (3.5)	103.0 (4.0)	92.5 (3.1)	74.6 (1.6)	87.7 (3.0)	84.8 (2.6)	98.8 (1.8)	131.7 (4.1)	94.1 (2.1)	75.4 (3.0)
10 µm + DS3b	43.9 (6.1)	71.8 (6.3)	60.1 (5.7)	79.0 (3.0)	86.5 (4.8)	70.3 (6.2)	87.5 (3.1)	117.6 (5.7)	80.1 (4.8)	103.0 (3.8)	97.6 (6.0)	98.2 (4.2)	68.7 (6.9)	99.9 (4.9)	95.4 (5.9)	97.1 (0.7)	93.1 (3.9)	101.9 (3.8)	100.7 (2.5)	101.9 (4.4)	98.8 (4.7)	101.3 (4.3)	96.8 (4.5)	101.6 (3.6)	103.3 (5.0)	101.6 (3.9)	104.4 (3.7)	105.4 (2.8)	101.2 (4.8)	83.7 (5.9)
10 µm polar + DS3b	27.6 (1.6)	50.6 (7.4)	37.0 (8.5)	61.0 (4.7)	72.8 (6.3)	43.6 (6.9)	64.4 (7.4)	109.9 (1.5)	62.3 (5.4)	97.7 (1.3)	82.3 (5.8)	92.5 (1.1)	44.8 (7.6)	92.0 (3.9)	84.9 (3.4)	86.7 (0.6)	90.9 (2.4)	94.3 (0.6)	89.5 (1.8)	96.7 (3.9)	92.9 (2.3)	94.0 (0.6)	83.2 (3.4)	92.2 (2.5)	95.1 (3.0)	91.6 (2.7)	97.1 (1.5)	100.6 (2.4)	96.0 (2.2)	87.5 (3.0)
5 µm + DS3b	34.3 (1.9)	61.8 (4.3)	49.0 (4.3)	69.7 (5.4)	77.5 (3.2)	58.3 (5.1)	78.7 (4.1)	108.7 (1.8)	70.4 (4.0)	99.8 (3.0)	89.5 (3.4)	95.4 (3.2)	59.6 (1.7)	96.3 (3.4)	88.5 (6.0)	92.4 (2.3)	87.7 (6.7)	98.8 (0.6)	94.1 (2.6)	96.6 (5.5)	96.5 (5.6)	98.0 (2.8)	89.5 (2.9)	94.4 (2.0)	100.8 (2.7)	95.8 (1.9)	99.0 (2.2)	101.3 (4.4)	99.8 (5.0)	81.9 (9.0)
45 µm + DS3b	46.6 (5.1)	76.4 (4.0)	66.5 (2.8)	89.8 (1.4)	90.6 (2.0)	73.9 (2.3)	86.7 (2.7)	108.5 (2.7)	84.8 (2.6)	101.0 (1.4)	96.1 (0.8)	99.5 (1.4)	74.1 (4.0)	99.4 (3.1)	89.9 (2.0)	93.8 (0.3)	82.5 (4.7)	104.8 (1.1)	98.1 (0.8)	95.8 (3.5)	97.8 (6.3)	98.8 (2.1)	95.1 (0.8)	99.2 (1.0)	101.8 (1.0)	100.3 (1.0)	101.8 (0.8)	100.8 (2.1)	94.3 (2.0)	74.1 (2.6)

Desorption solvents compositions: DS1a = isopropanol/water/formic acid (80/19.9/0.1, v/v); DS1n = isopropanol/water (80/20, v/v); DS1b = isopropanol/water/ammonium hydroxide (80/19.9/0.1, v/v); DS2a = acetonitrile/water/formic acid (80/19.9/0.1, v/v); DS2n = acetonitrile/water (80/20, v/v); DS2b = acetonitrile/water/ammonium hydroxide (80/19.9/0.1, v/v); DS3a = methanol/water/formic acid (80/19.9/0.1, v/v); DS3n = methanol/water (80/20, v/v); DS3b = methanol/water/ammonium hydroxide (80/19.9/0.1, v/v).

Table S4. Essential physicochemical proprieties of the analysed substances. *Substances arranged by retention order.*

substance	monoisotopic mass [Da] [PubChem 2.1][1]	logP [XLogP3.0][1]	logP [ALOGPS][2,3]	logP [ChemAxon][2,3]	logP [ACD/Labs][4]	logD ph=7.4 [ACD/Labs][4]	polar surface area [Å^2] [Cactvs 3.4.6.11][1]	polar surface area [Å^2] [ChemAxon][2,3]	polar surface area [Å^2] [ACD/Labs][4]	polarizability [Å3] [ChemAxon][2,3]	polarizability [Å3] [ACD/LABS][4]	pKa (strongest acidic) [ChemAxon][2,3]	pKa (strongest basic) [ChemAxon][2,3]	H acceptors [Cactvs 3.4.6.11][1]	H donors [Cactvs 3.4.6.11][1]	H acceptors [ChemAxon][2,3]	H donors [ChemAxon][2,3]	H acceptors [ACD/Labs][4]	H donors [ACD/Labs][4]
fenoterol	303.147058	2.0	1.36	1.47	0.89	-0.45	93.0	92.95	93	31.75	33.7	8.85	9.63	5	5	5	5	5	5
carteolol	292.178693	1.0	1.05	1.42	1.35	-0.24	70.6	70.59	71	32.79	32.3	13.41	9.76	4	3	4	3	5	3
oxycodone	315.147058	1.2	1.04	1.03	1.67	0.45	59.0	59.00	59	32.79	33.0	13.57	8.77	5	1	5	1	5	1
hydrocodone	299.152144	2.2	2.13	1.96	1.83	0.17	38.8	38.77	39	32.05	32.3	18.00	8.61	4	0	4	0	4	0
ketamine	237.092042	2.2	2.69	3.35	2.18	2.07	29.1	29.10	29	24.97	26.1	18.78	7.45	2	1	2	1	2	1
remifentanil acid	362.184172	-0.7	N/A	N/A	1.58	-1.14	87.2	N/A	87	N/A	38.1	N/A	N/A	6	1	N/A	N/A	7	1
metoprolol	267.183444	1.9	1.80	1.76	1.79	-0.25	50.7	50.72	51	31.90	30.6	14.09	9.67	4	2	4	2	4	2
6-acetylcodeine	341.162708	1.7	N/A	N/A	2.09	1.12	48.0	N/A	48	N/A	36.6	N/A	N/A	5	0	N/A	N/A	5	0
methylphenidate	233.141579	0.2	1.47	2.25	2.55	0.26	38.3	38.33	38	26.21	26.4	N/A	9.09	3	1	2	1	3	1
zolpidem	307.168462	2.5	3.15	3.02	3.07	3.06	37.6	37.61	38	35.06	37.1	N/A	5.65	2	0	2	0	4	0
cocaine	303.147058	2.3	1.97	2.28	3.08	1.22	55.8	55.84	56	32.36	32.2	N/A	8.85	5	0	3	0	5	0
LSD	323.199762	3.0	3.30	2.28	2.74	2.60	39.3	39.34	39	37.54	38.5	17.02	7.98	2	1	2	1	4	1
melatonin	232.121178	0.8	1.42	1.15	0.96	1.74	54.1	54.12	54	25.65	26.8	15.80	-1.60	2	2	2	2	4	2
bisoprolol	325.225308	1.9	2.30	2.20	2.14	0.12	60.0	59.95	60	38.50	36.7	14.09	9.67	5	2	5	2	5	2
phencyclidine	243.198700	3.6	5.31	4.49	4.89	3.66	3.2	3.24	3	29.66	30.4	N/A	10.56	1	0	1	0	1	0
cortisol	362.209324	1.6	1.79	1.28	1.43	1.66	94.8	94.83	95	39.45	37.9	12.59	-2.80	5	3	5	3	5	3
buprenorphine	467.303559	5.0	4.53	3.55	3.43	3.48	62.2	62.16	62	53.11	52.1	7.50	12.54	5	2	5	2	5	2
alprazolam	308.082874	2.1	2.23	2.37	2.50	2.63	43.1	43.07	43	32.22	35.0	18.30	5.08	3	0	3	0	4	0
anastrozole	293.164046	2.1	2.31	3.03	0.97	2.68	78.3	78.29	78	31.97	35.7	N/A	2.00	4	0	4	0	5	0
methadone	309.209264	3.9	4.14	5.01	4.20	2.80	20.3	20.31	20	36.28	38.0	19.79	9.12	2	0	2	0	2	0
11-deoxycortisol	346.214409	2.5	2.97	2.58	2.74	2.67	74.6	74.60	75	38.86	37.3	12.59	-3.30	4	2	4	2	4	2
boldenone	286.193280	3.5	3.08	3.36	3.50	3.31	37.3	37.30	37	33.26	32.9	18.86	-0.88	2	1	2	1	2	1
clonazepam	315.041069	2.4	2.76	3.15	2.34	2.53	87.3	87.28	87	29.59	32.2	11.89	1.86	4	1	4	1	6	1
agomelatine	243.125929	2.7	2.83	2.04	2.27	3.02	38.3	38.33	38	27.18	29.0	15.96	-0.94	2	1	2	1	3	1
methandienone	300.208930	3.6	3.55	3.64	4.04	3.76	37.3	37.30	37	35.09	34.8	18.86	-0.53	2	1	2	1	2	1
flunitrazepam	313.086269	2.1	2.20	2.55	1.44	2.03	78.5	78.49	78	29.60	32.4	N/A	1.70	5	0	4	0	6	0
androstenedione	286.193280	2.7	2.93	3.93	2.90	2.90	34.1	34.14	34	33.20	32.4	19.03	-4.80	2	0	2	0	2	0
canrenone	340.203845	2.7	2.79	3.60	2.99	2.50	43.4	43.37	43	38.67	37.8	N/A	-4.80	3	0	2	0	3	0
progesterone	314.224580	3.9	3.58	4.15	4.04	3.72	34.1	34.14	34	37.26	36.1	18.47	-4.80	2	0	2	0	2	0
THC-COOH	344.198759	6.3	5.24	5.14	6.21	3.07	66.8	66.76	67	39.41	38.4	4.21	-4.90	4	2	4	2	4	2

References:

1 PubChem database, https://pubchem.ncbi.nlm.nih.gov/, (accessed March 31, 2021).

2 DrugBank database, https://www.drugbank.com/, (accessed March 31, 2021).

3 The Human Metabolome Database, https://hmdb.ca/, (accessed March 31, 2021).

4 ChemSpider database, http://www.chemspider.com/, (accessed March 31, 2021.

Table S5. List of reference standards in alphabetical order.

substance	reference standard	grade	manufacturer
(-)-11-nor-9-carboxy-delta9-THC (THC-COOH)	MeOH solution 1 mg/mL	CRM (primary standard)	LGC
6-acetylcodeine	ACN solution 1 mg/mL	CRM (primary standard)	Cerillant
11-deoxycortisol	MeOH solution 1 mg/mL	CRM (primary standard)	Cerillant
agomelatine	MeOH solution from powder 1 mg/mL	N/A	TRC
alprazolam	MeOH solution 1 mg/mL	CRM (primary standard)	LGC
anastrozole	MeOH solution from powder 1 mg/mL	reference standard (100%)	LGC
androstenedione	ACN solution 1 mg/mL	CRM (primary standard)	Cerillant
bisoprolol	MeOH solution 1 mg/mL	CRM (primary standard)	LGC
boldenone	MeOH solution from powder 1 mg/mL	analytical standard (≥ 98%)	VETRANAL™ (Sigma-Aldrich)
buprenorphine	MeOH solution 1 mg/mL	CRM (primary standard)	Cerillant
canrenone	MeOH solution from powder 1 mg/mL	HPLC (≥ 97%)	Sigma
carteolol	MeOH solution from powder 1 mg/mL	USP reference standard (100%)	USP
clonazepam	MeOH solution 1 mg/mL	CRM (primary standard)	Cerillant
cocaine	ACN solution 1 mg/mL	CRM (primary standard)	LGC
cortisol	MeOH solution from powder 1 mg/mL	N/A	N/A
fenoterol	MeOH solution from powder 1 mg/mL	reference standard (99.9%)	LGC
flunitrazepam	MeOH solution 1 mg/mL	CRM (primary standard)	LGC
hydrocodone	MeOH solution 1 mg/mL	CRM (primary standard)	Cerillant
ketamine	MeOH solution 1 mg/mL	CRM (primary standard)	LGC
lysergic acid diethylamide (LSD)	ACN solution 1 mg/mL	CRM (primary standard)	LGC
melatonin	MeOH solution from powder 1 mg/mL	TLC (≥98%)	Sigma
methandienone	1,2-dimethoxyethane solution 1 mg/mL	CRM (primary standard)	Cerillant
methadone	MeOH solution 1 mg/mL	CRM (primary standard)	Cerillant

methylphenidate	MeOH solution 1 mg/mL	CRM (primary standard)	LGC
metoprolol	MeOH solution 1 mg/mL	CRM (primary standard)	LGC
oxycodone	MeOH solution 1 mg/mL	CRM (primary standard)	LGC
phencyclidine	MeOH solution 1 mg/mL	CRM (primary standard)	LGC
progesterone	MeOH solution from powder 1 mg/mL	N/A	N/A
remifentanil acid	ACN solution 100 µg/mL	CRM (primary standard)	Cerillant
zolpidem	MeOH solution 1 mg/mL	CRM (primary standard)	LGC
deuterium-labelled internal standards			
alprazolam D_5	MeOH solution 100 µg/mL	CRM (primary standard)	Cerillant
cocaine D_3	ACN solution 100 µg/mL	CRM (primary standard)	Cerillant
oxycodone D_3	MeOH solution 100 µg/mL	CRM (primary standard)	Cerillant
(-)-11-nor-9-carboxy-delta9-THC D_3 (THC-COOH D_3)	MeOH solution 100 µg/mL	CRM (primary standard)	Cerillant

Table S6. Monitored precursor – product ion(s) transitions. *Substances arranged by retention order.*

substance	retention time [min]	precursor ion [m/z]	product ions [m/z]:		
			1	2	3
fenoterol	4.156	304.00	107.15	135.15	286.10
carteolol	4.499	293.00	237.15	202.15	74.10
oxycodone D_3	4.532	319.00	301.20	244.10	259.20
oxycodone	4.567	316.10	298.20	241.20	256.10
hydrocodone	4.914	300.00	199.15	171.10	128.15
ketamine	5.711	237.90	125.05	220.10	207.15
remifentanil acid	6.755	363.00	113.10	146.20	214.15
metoprolol	6.499	267.90	116.15	74.15	72.10
6-acetylcodeine	6.911	342.00	225.15	165.15	197.20
methylphenidate	6.765	234.00	84.15	56.10	91.10
zolpidem	7.537	307.90	235.15	236.20	263.20
cocaine	7.501	303.90	182.20	82.10	105.10
cocaine D_3	7.503	307.00	185.20	77.10	85.20
LSD	7.844	323.90	223.20	208.15	207.10
melatonin	8.048	232.90	174.20	130.15	159.10
bisoprolol	8.018	326.20	116.20	74.05	72.10
phencyclidine	8.828	244.00	91.05	86.10	159.20
cortisol	9.490	363.20	121.00	327.20	105.00
buprenorphine	9.891	468.10	55.15	396.25	414.25
alprazolam D_5	11.420	314.10	210.20	286.10	279.25
alprazolam	11.484	308.90	205.15	281.15	274.10
anastrozole	11.506	294.00	225.20	210.20	115.05
methadone	11.622	310.00	265.15	105.10	77.05
11-deoxycortisol	11.617	347.15	97.10	109.05	79.05
boldenone	12.042	287.00	121.20	135.20	77.15
clonazepam	12.062	316.00	270.10	214.05	207.15
agomelatine	12.210	244.00	185.20	170.10	141.10
methandienone	12.691	301.20	121.05	149.25	77.00
flunitrazepam	12.851	313.90	268.15	239.10	183.10
androstenedione	14.570	287.30	97.05	109.05	78.95
canrenone	15.153	341.00	107.15	187.25	235.15
progesterone	17.880	315.00	97.10	109.10	297.30
THC-COOH D_3	19.956	348.00	330.20	302.25	196.25
THC-COOH	19.982	345.00	327.10	299.25	193.30
